# Dim Light at Night Induced Neurodegeneration and Ameliorative Effect of Curcumin

**DOI:** 10.3390/cells9092093

**Published:** 2020-09-13

**Authors:** Dhondup Namgyal, Kumari Chandan, Armiya Sultan, Mehreen Aftab, Sher Ali, Rachna Mehta, Hamed A. El-Serehy, Fahad A. Al-Misned, Maryam Sarwat

**Affiliations:** 1 Amity Institute of Neuropsychology and Neuroscience, Amity University, Noida UP 201303, India; dhonamdhonam@gmail.com (D.N.); rmehta2@amity.edu (R.M.); 2 Amity Institute of Pharmacy, Amity University, Noida UP 201303, India; kumarichandandu@gmail.com; 3 Center for Interdisciplinary Research in Basic Sciences, Jamia Millia Islamia, New Delhi 110025, India; armia86bms@gmail.com; 4 Amity Institute of Molecular Medicine and Stem Cell Research, Amity University, Noida UP 201303, India; mehreen_aftab2004@yahoo.com; 5 School of Basic Sciences and Research, Department of Life Sciences, Sharda University, Greater Noida, Uttar Pradesh 201310, India; sher.ali@sharda.ac.in; 6 Department of Zoology, College of Science, King Saud University, Riyadh l1451, Saudi Arabia; helserehy@ksu.edu.sa (H.A.E.-S.); almisned@ksu.edu.sa (F.A.A.-M.)

**Keywords:** lipid peroxidation, oxidative stress, diurnal rhythm, brain-derived neurotrophic factor, curcumin, neurodegeneration, neurogenesis, neuroprotection, microRNA

## Abstract

It is a well-known fact that following a proper routine light/dark or diurnal rhythm controls almost all biological processes. With the introduction of modern lighting and artificial illumination systems, continuous exposure to light at night may lead to the disruption of diurnal rhythm. However, the effect of light during the night on brain anatomy, physiology, and human body functions is less explored and poorly understood. In this study, we have evaluated the effect of exposure to dim light (5 lux) at night (dLAN) on Swiss Albino mice over a duration of three consecutive weeks. Results have revealed that exposure to dLAN led to an impairment of cognitive and non-cognitive behaviour, oxidative stress–mediated elevation of lipid peroxidation, and reduction of superoxide dismutase and catalase activity. It also led to the downregulation of hippocampal proteins (BDNF, Synapsin II and DCX) at both protein and mRNA level. Additionally, there was downregulation of *CREB* and *SIRT1* mRNAs and neurodegeneration-associated miRNA21a-5p and miRNA34a-5p. The pyramidal and cortical neurons started showing pyknotic and chromatolysis characteristics. However, a dose of curcumin administered to the mice positively modulated these parameters in our experimental animals. We proposed the modulatory role of curcumin in addressing the deleterious effects of dLAN.

## 1. Introduction

Modernization and advancement of technology has brought about tremendous benefits and improvement in the health care system and has primarily led to an elongated human lifespan. On the flip side, it has also made us more prone to behavioural impairment and age-related neurodegenerative diseases [1]. The natural cycle of day (light) and night (dark) had accompanied the evolution of animal and plants since the beginning of life on earth. This cycle has led to the development of a diurnal rhythm in each living organism; based on this cycle, our physiological processes are adapting and changing continuously. It is a well-established fact that the daily light/dark cycle or diurnal rhythm controls almost all biological processes including learning, memory, mood regulation, and rest–activity and sleep–wake cycles [2,3,4].

Today, most of the people living in urban areas are unable to see the natural celestial light on a clear night as they are often confined within the domains of artificial lighting systems at home or workplaces. The 24 × 7 occupational work system in our society has become a routine feature, and people work continuously and socialize round the clock. However, people are gradually realizing the ill effects of night-time light exposure and considering it as an environmental perturbation [5,6]. Recent studies have indicated that alteration of daily light dark cycle contributes to the development of many pathophysiological conditions such as cardiovascular disease [7,8], metabolic syndrome [9,10], sleep disorders [11], aging [12], neurodegenerative disorders [13,14,15], mood disorders [16], and cognitive impairment [17]. Exposure to artificial lighting during the night (dim light at night (dLAN)) and shift working are reported to interfere with proper physiological functioning of the brain and body [18,19,20,21]. Further, it has been highlighted that exposure to dLAN (5 lux) reduces the expression of proteins (such as brain-derived neurotrophic factor (BDNF)) required for the growth and functioning of nerve cells. This in turn negatively impacts our neuroplasticity, learning, and memory [22]; increases susceptibility to stress-induced oxidative damage [23]; and impairs the process of spatial learning and memory retention function [24]. Apart from BDNF, synapsin II, doublecortin (DXC), and cyclic adenosine monophosphate response binding (cAMP response-element binding protein (CREB)) and SIRT1 proteins are also associated with ‘neurogenesis’ and ‘synaptogenesis’. These two processes are the components of the major pathways involved in the brain development. Impairment in any of these pathways can lead to age-related neurodegeneration. Moreover, exposure to constant light for three weeks in mice have reported to reduce hippocampal neurogenesis as seen by reduced BrdU-positive cells [25]. Therefore, our first hypothesis is based on the premise that exposure to dLAN (5 lux) for three consecutive weeks could lead to neurodegeneration of the hippocampal regions modulated by oxidative stress, and this neurodegeneration could lead to an impairment of hippocampal dependent memory and learning functions.

Curcumin is a natural polyphenol molecule whose therapeutic properties have been explored in neuropsychiatric and neurodegenerative in vitro and in vivo models [26,27]. Even though the bioavailability of curcumin is restricted by the blood–brain barrier, many researchers had reported its highly effective therapeutic ability in the brain disorders including Alzheimer’s, Parkinson’s, and Huntington’s diseases; multiple sclerosis; depression; and schizophrenia [28,29,30,31]. With advancement in the field of researcher, the bioavailability of curcumin has been improved tremendously through the development of nano-particle-encapsulated curcumin administration [32,33,34,35]. In addition, its oral administration has been found to improve the learning and memory in animal models [36,37,38,39,40,41]. In our previous study, we saw that curcumin treatment effectively promoted neurogenesis in mice and reverses the adverse effects of Cd-induced neurotoxicity [42].

**Figure 1:**
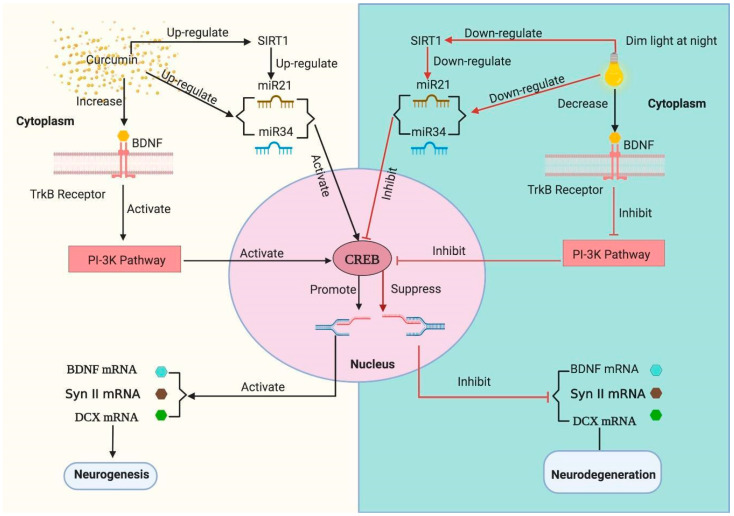
In the mice exposed to dim light (5 lux) at night (dLAN), reduction of the brain-derived neurotrophic factor (BDNF) protein led to down-regulation of tyrosine kinase B neurotrophic receptor (TrkB). This leads to down-regulation of the phosphoinositide 3-kinase (PI3K) pathway and deactivation of the transcription factor cyclic AMP response-element binding protein (CREB). Since CREB is responsible for the production of BDNF, Synapsin II, and DCX proteins, inhibition of these transcripts leads to neurodegeneration. Further, in the dLAN-exposed group, the reduction of the SIRT1 gene down-regulates the miRNA21a-5p and miRNA34a-5p which inhibits the CREB pathway. Curcumin treatment increases the hippocampal BDNF protein which leads to up-regulation of TrkB neurotropic receptor, activation of PI3K pathway and transcription of CREB. The activation of CREB transcript leads to neurogenesis and improvement of learning and memory. The expression of miRNA21a-5p and miRNA34a-5p also increased in the curcumin treated groups. which leads to up-regulation of the downstream products including TrkB and CREB.

These reports strongly support the neuroprotective effects of curcumin, yet the role of curcumin in dLAN-induced neurodegeneration has remained unexplored thus far. Therefore, we propound a hypothesis (Figure 1) that curcumin may improve the cognitive and non-cognitive behaviour of mice exposed to dLAN (5 lux) for three consecutive weeks through reduction of oxidative stress and increased production of hippocampal proteins (BDNF, Synapsin II, DCX, CREB (Cyclic AMP response element-binding protein) and SIRT1 (nicotinamide-adenine dinucleotide-dependent deacetylase)] which are essentially involved in the neurogenesis process.

## 2. Materials and Methods

### 2.1. Biochemical Reagents and Instruments

Curcumin extract (90%) was purchased from Sisco Research Laboratories (SRL) Pvt. Ltd., (Mumbai, Maharashtra, India). High Capacity cDNA kit, phosphate buffer saline (PBS), formaldehyde, 5,5-dithiobis (2-nitrobenzoic acid) (DTNB), trichloroacetic acid, thiobarbituric acid (TBA), butanol, nitroblue tetrazolium (NBT), disodium hydrogen phosphate, sodium citrate, and hydrogen peroxide (H_2_O_2_) were obtained from Thermo Fisher Scientific (Waltham, MA, USA). Mouse brain-derived neurotrophic factor (BDNF) ELISA kit and mouse CREB ELISA kit were purchased from Ray Biotech (Georgia, GA, USA). Mouse Synapsin ELISA kit and mouse DCX ELISA kit were purchased from Gentaur (Kampenhout, Belgium). RNA Isolation Reagents and TRIzol were purchased from Sigma Aldrich (St. Louis, MO, USA). SYBR Green was purchased from Applied Biosystems, MA, USA. NanoDrop (NanoDrop Technologies, Wilmington, DE, USA), Polytron tissue homogenizer (Thomas Scientific, Swedesboro, NJ, USA), ELISA reader (Trans Asia Pvt. Ltd., Mumbai, Maharashtra, India), and UV spectrometer (Perkin Elmer, Waltham, MA, USA) were used during the conduction of experimental procedures.

### 2.2. Animals and Experimental Conditions

Swiss male albino mice (*n* = 30, age = four weeks, weight = 25–30 g) were used as model organisms in the current study. The mice were procured from the Animal House of Amity University, Noida, India. All experimental procedures were approved by the Institutional Animal Care and Ethical Committee of Amity University (CPCSEA/IAEC/AIP/2019/01/25), and the animals were maintained in accordance with the recommendations of the committee for Control and Supervision of Experiments on Animal (CPCSEA), India. All mice were individually kept in propylene cages (dimension: 33 cm × 19 cm × 14 cm) at an optimum temperature of 22–24 °C under standard light–dark cycle (LD; 12:12 light (~150 lux)/dark (0 lux)) for three weeks of experimentation. Mice were fed with Harlan Tekla 8640 food (Madison, WI, USA) and filtered tap water ad libitum. All experimental procedures were approved by the Institutional Animal Care and Ethical Committee of Amity University, and the animals were maintained in accordance with the recommendations of the Committee for the Purpose of Control and Supervision of Experiments and Animals (CPCSEA/IA/EC/AIP/2017/03/02), India. After one week of acclimation, mice were randomly distributed to five groups and were transferred to cabinets kept either under normal LD cycle (12:12 light (~150 lux)/dark (0 lux)) or under a light/dim light (dLAN) cycle (12:12 light (~150 lux)/dim light (~5 lux)). Daytime lighting was provided with white LEDs fixed on the walls of cabinets, and dim light conditions were created using a flexible strip of cool white LED bulbs kept above the rack on which the mice cages were placed. The intensity and consistency of lighting was measured using a lux meter.

### 2.3. Experimental Design and Treatment

Curcumin extract (90%) was dissolved in 1% carboxymethyl cellulose (CMC) for administration in mice. Mice were divided into the following five groups (*n* = 6 per group):

1. LD control group (12:12 light (~150 lux)/dark (~0 lux), 1% CMC);
2. dLAN control group (12:12 light (~150 lux)/dim light (~5 lux), 1% CMC);
3. dLAN + 50 mg/kg curcumin (dLAN + Cur50);
4. dLAN + 100 mg/kg curcumin (dLAN + Cur100);
5. dLAN + 150 mg/kg curcumin (dLAN + Cur150).

**Figure 2:**
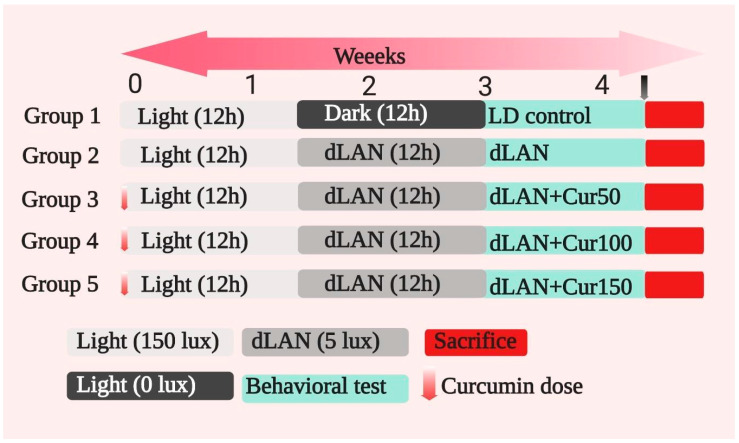
Details of experiment design. Total 30 Swiss Albino mice (age = four weeks, weight = 25–30 g) were used in the present study. The mice were divided into following groups and treated with different concentrations of curcumin (50, 100, and 150 mg/kg). The details of each group are as follows:

The details of experimental design and curcumin treatment are depicted in Figure 2 and Table S1. Carboxymethyl cellulose (CMC) and different concentrations of curcumin were orally administered to mice.

1. LD control group (12:12 light (~150 lux)/dark (~0 lux), 1% CMC) (*n* = 6);
2. dLAN exposed group (12:12 light (~150 lux)/dim light (~5 lux), 1% CMC) (*n* = 6);
3. dLAN + 50 mg/kg curcumin (dLAN + Cur50) (*n* = 6);
4. dLAN + 100 mg/kg curcumin (dLAN + Cur100) (*n* = 6);
5. dLAN + 150 mg/kg curcumin (dLAN + Cur150) (*n* = 6).

LD: light/dark; dLAN: dim light at night; CMC: carboxymethyl cellulose; Cur50: curcumin 50 mg/kg; Cur100: curcumin 100 mg/kg; Cur150: curcumin 150 mg/kg.

### 2.4. Behaviour Studies

The experimental data of the behavioural tests were collected in the evening as mice are nocturnal animals and they remain active during dark phase. In order to have unbiased recording of results, we have taken the help of a person who is blind to the experimental details.

#### 2.4.1. Open-Field Test

After three weeks of treatment, an open-field test (OFT) was performed to study the locomotor activity and anxiety-like behaviour of mice in the evening (18:00) before the onset of dark phase. The OFT apparatus was kept in a medially lit room (~20 lux) consisting of a square arena (area 40 cm × 40 cm), divided into four centre squares and 16 peripheral squares. A video recording device was placed above the arena to record the locomotor activity and behaviour of the mice. Animals were individually kept in the centre of the OFT apparatus and were given free rein to explore the area for 10 min. During this time window, multiple parameters measured were as follows:

1. Total number of crossings from one square to another (locomotor activity)—NLC;
2. Crossing in the centre square (stress and anxiety level)—CSE;
3. Time spent in the centre square (stress and anxiety level)—DCS;
4. Rearing frequency (number of times the animal stood on their hind paws) (anxiety-like behaviour)—RR.

Line crossed was considered only when an animal entered another square with all four of its paws. In between each test, OFT apparatus was periodically cleaned with a 10% ethanol and water in order to remove any odour left by the other mice [43].

#### 2.4.2. The Novel Object Recognition Test

Novel object recognition test is used to study the recognition memory of mice by using two familiar objects in the trial test and subsequently replacing one familiar object with a novel object. Recognition memory was assessed using the object recognition index by measuring the time duration spent in the exploration of the novel object as compared to the familiar object (T2/T1). The apparatus is comprised of a square-shaped open field box made of black plexiglass having dimensions of 40 cm × 40 cm. Moreover, it has two familiar objects located on the opposite side of the starting point. All the mice were allowed to explore these two familiar and identical objects (cylinders) in the open field apparatus for a time frame of five minutes. This exposure represents the learning period. Each mouse went twice daily (both morning and evening) through this five-minute learning period for two consecutive days. After two days, the mice were individually allowed to explore the familiar (cylinder) and the novel object (cuboid) as part of the learning period protocol in the evening (18:00) before the onset of dark phase. The time spent in exploring the familiar object (T1) and novel object (T2) was recorded for the computation and analysis of recognition memory index [44].

#### 2.4.3. Morris Water Maze Test

Cognitive functions such as spatial learning and retention memory were assessed using the Morris Water Maze (MWM) test [45]. All the mice were released in a large circular pool (130 cm in diameter and 60 cm high) which was filled with warm and cloudy water to a depth of 30 cm. A target platform was kept hidden at a depth of 2 cm below the surface of the water in the first quadrant. We measured the acquisition of memory retention and cognitive functions for a period of five days. In a set of four trials per day, each mouse was randomly released in the pool facing a wall at one of the four points (east, west, south, or north) and given up to 90 s to find the invisible platform. Once the mouse was able to locate the platform or be guided to the platform after 90 s, the mice were permitted to remain on the platform for 10 s. The total time taken to find the hidden platform and the time spent in the correct platform quadrant was measured. Using a video recording device, the frequency with which the mice were able to locate the hidden platform and the average time duration spent in the correct platform quadrant was assessed.

#### 2.4.4. Tissue Sample Collection

Once the behavioural tests were completed, the mice were anesthetized using sodium thiopental (50 mg/kg) and sacrificed in the morning time (09:00–11:00) in order to avoid the interference of stress hormones. The skulls were opened, and the hippocampus was dissected on ice-cold surgical plates. Hippocampus tissue samples from different groups were homogenized in phosphate buffer saline (PBS, pH 7.4) and centrifuged at 10,000 rpm for 15 min. Supernatants were collected for biochemical and protein analyses.

### 2.5. Estimation of Oxidative Stress

#### 2.5.1. Lipid Peroxidation

Degree of lipid peroxidation in the hippocampus of different mice groups was determined quantitatively using a method described by Wills [46]. Samples collected were mixed with 1 mL of 10% trichloroacetic acid and 1 mL of 0.67% thiobarbituric acid and heated in a boiling water bath for 15 min. Butanol (2:1 *v/v*) was added to the solution. Level of malondialdehyde (MDA) was assessed by reaction with thiobarbituric (TBA) acid at 532 nm using a UV-spectrophotometer. Values were derived using the molar extinction coefficient of MDA-TBA adduct at 532 nm that is 155 (nM/cm).

#### 2.5.2. Superoxide Dismutase Activity

Superoxide dismutase activity (SOD) was assessed by the nitroblue tetrazolium (NBT) method based on the principle that NBT undergoes a photo-reduction (which is blue-coloured formazan) when exposed to light by superoxide radicals. It competes with the enzyme SOD for superoxide anions. With the presence of SOD in the reaction mixture, NBT produces a lesser quantity of coloured complex as compared to control. Hippocampal tissue homogenate (500 μL) was mixed with chloroform (300 μL) and ethanol (500 μL). The mixture was centrifuged at 18,000× *g* for 30 min, and 50 μL of supernatant was mixed with 900 μL of SOD reagent (0.1 mmol/L xanthine, 0.1 mmol/L EDTA, 50 mg bovine serum albumin, 25 mmol/L NBT and 40 mmol/L Na_2_CO_3_) (pH 10.2). Further, 25 units of xanthine oxidase was added to the mixture and incubated for 20 min at 25 °C. The reaction was stopped by adding 1 mL of CuCl_2_ (0.8 mmol/L), and absorbance was recorded at 560 nm [47].

#### 2.5.3. Catalase Activity

Catalase enzyme activity was measured at 240 nm using a spectrophotometer. Then, 1 mL of hippocampal tissue homogenate was briefly poured into a test tube containing 1.9 mL of phosphate buffer (PBS) (50 mM, pH 7.4). Furthermore, 1 mL of 30 mM H_2_O_2_ was added to the mixture to initiate the reaction. A mixture of 2.9 mL PBS and 1 mL H_2_O_2_ without hippocampal tissue homogenate was considered as the blank. H_2_O_2_ decomposition resulted in a reduction of absorbance, recorded at 240 nm against the blank. The unit of catalase activity was expressed as the amount of enzyme that decomposes 1 μM of H_2_O_2_ per minute at 25 °C using the molar coefficient of 43.6 M/cm. This activity was expressed in terms of unit/mg proteins [48].

### 2.6. Morphometric and Histopathological Analyses

For histological analysis, mice were randomly selected and sacrificed in the morning time (09:00–11:00) in order to avoid the interference of stress hormones. Dissected brains of the mice were fixed in methanol/chloroform/acetic acid solution (6:3:1) and stored in 10% formaldehyde. Subsequently, brain tissues were dehydrated in ethanol. Thereafter, tissues were clarified using xylene and were embedded in Paraplast Plus. Paraffin-embedded coronal sections of 3-μm thickness were cut with a microtome and stained with hematoxylin (H) and eosin (E) before being mounted onto saline-coated slides for microscopic examination at 100× [49]. Morphometry was carried out by analysing CA1, CA2, and CA3 regions of the hippocampal pyramidal cells and cerebral cortex. From each brain sample, five sections were stained for analyses. The neurotoxic effect of dLAN and neuroprotective effect of curcumin on neuronal cells (hippocampal pyramidal cells and necrotic eosinophilic neurons) and biological processes (pyknosis and chromatolysis) were qualitatively analysed using an Olympus BX43 light microscope (Olympus, Tokyo, Japan). The presence of hypereosinophilic cytoplasm or pyknotic nuclei was used to identify non-viable neurons.

### 2.7. Hippocampus Protein Estimation

For evaluation of the effect of dLAN and the counter effect of curcumin on the levels of hippocampal proteins, we selected three proteins—namely, BDNF, Synapsin II, and DCX. All three selected proteins are closely related to neurodegeneration and neurogenesis processes. Mice (*n* = 6) from each group were anaesthetised and sacrificed after the MWM test. The hippocampus was dissected and weighed, and the level of proteins (namely BDNF, Synapsin II, and DCX) were measured using specific ELISA kits. The hippocampus tissue was weighed, and 300 μL of lysis buffer was added to each sample. Subsequently, the samples were homogenized for 30 s and centrifuged at 10,000 rpm for 15 min at 4 °C. All samples were assayed in triplicate. Absorbance was measured at 450 nm with an ELISA plate reader (Trans Asia Pvt. Ltd., Mumbai, Maharashtra, India). Total protein concentration was estimated using the Bio-Rad (Hercules, CA, USA) protein quantification protocol. The concentration of each protein sample was calculated by plotting the absorbance values on a standard curve as generated by the assay.

### 2.8. RNA Extraction and cDNA Synthesis

From the frozen samples of mice hippocampal tissue, total RNA was extracted, following the protocol of Sarwat and Naqvi [50]. The extraction was carried out by the TRIzol reagent method employing the total RNA isolation reagent (Sigma Aldrich, St. Louis, MO, USA). RNA was dissolved in 30 µL nuclease-free water. The isolated RNA products were analyzed by electrophoresis on a 1% agarose gel. RNA was quantified with a NanoDrop (NanoDrop Technologies, Wilmington, DE, USA) and the concentration of RNA was calculated from the optical density at 260 nm. The purity of RNA was determined by 260 nm/280 nm of absorbance. Further, cDNA was synthesised from total RNA templates using the High Capacity cDNA kit (Thermo Fisher Scientific, Waltham, MA, USA). RNA was reverse transcribed using 1000 ng of total RNA and 10X RT primers following the manufacturer’s instructions. The miRNAs were reverse transcribed using 1000 ng of total RNA and a pool of miRNA specific stem-loop primers according to manufacturer’s instructions. The cDNA synthesized was stored in −20 °C for later use as a template for RT-PCR.

#### 2.8.1. Quantitative RT-PCR

Quantitative real-time polymerase chain reaction (qRT-PCR) of each gene was done in the treated (dLAN, dLAN + curcumin) as well untreated control (LD). According to manufacturer’s instructions, 1 µL of the cDNA sample was employed for the qRT-PCR using gene-specific forward and reverse primers (Table S2) and SYBR Green master mix (Applied Biosystems, Foster City, CA, USA). All the samples were run in a StepOne™ Real-Time PCR System (Applied Biosystems, Foster City, CA, USA) using a program wherein the melting curve analysis ascertains single product formation. β-actin was run along with all individual genes and was used as a reference control to normalize the gene expression. PCR reaction was carried out for 10 min at 95 °C, followed by 45 cycles at 95 °C for 15 s and 60 °C for 1 min. All experiments were run in triplicate along with negative and positive controls. The change in relative gene expression fold for each sample including normal controls was calculated on the basis of the threshold cycle (CT) value calculated using the formula as relative quantification (RQ) = 2^−ΔΔCT^.

#### 2.8.2. miRNA Profiling in Brain Tissue Using SYBR Green

RT-PCR was performed separately for miR-21a-5p and miR-34a-5p with the corresponding normal controls using miRNA-specific forward and reverse primers (Table S3) and SYBR Green master mix (Applied Biosystem, Foster City, CA, USA). snRNA U6 was used as a reference control. Default threshold settings were used to ascertain the threshold cycle. The same concentration of cDNA was used for all miRNA analyses in order to maintain the same level of efficiency. PCR reaction was carried out for 10 min at 95 °C, followed by 45 cycles at 95 °C for 15 s and 60 °C for 1 min. All reactions were run in triplicate along with negative and positive controls. The change in relative miRNA expression fold for each sample including normal controls was calculated on the basis of CT value calculated using the formula as: Relative Quantification (RQ) = 2^−ΔΔCT^.

### 2.9. Statistical Analyses

Values are represented as mean ± standard deviation. The non-parametric *t*-test was employed to compare the results between LD and dLAN groups. Non-parametric one-way ANOVA test was employed to compare the behaviour result between dLAN vs. dLAN treatment groups. Two-way ANOVA test was employed to compare the biochemical, proteins and mRNA expression results between dLAN vs. dLAN + Cur50, dLAN + Cur100, and dLAN + Cur150 (described in the respective tables). All the data was analysed in GraphPad Prism-8 software. The values of *p < 0.05* represent a statistically significant difference between the groups.

## 3. Results

### 3.1. Curcumin Improved the Locomotor Activity and Anxiety-Like Behaviour in Mice Exposed to dLAN

Table 1 and Table 2 represent averages of open field test (OFT) with the following parameters: number of lines crossed (NLC), centre square entries (CSE), duration in the centre square (DCS), and rearing frequency (RR) in LD control, dLAN exposed, and dLAN treated (dLAN + Cur50, dLAN + Cur100 and dLAN + Cur150) groups of mice. Comparative results showed that averages of NLC (t = 19.27, *p* < 0.001), CSE (t = 5.549, *p* < 0.01), DCS (t = 11.45, *p* < 0.001), and RR (t = 19.36, *p* < 0.001) in mice exposed to dLAN were significantly lower as compared to LD control mice (Table 1). These findings reflect decreased locomotor activity and increased anxiety-like behaviour in mice exposed to dLAN and testify that continuous dLAN exposure leads to impairment of locomotor activity and increases the susceptibility of stress. In order to check the modulatory effect of curcumin on these bio-behavioural parameters, mice exposed to dLAN were treated with increasing concentrations (50, 100 and 150 mg/kg body weight) of curcumin. Results showed that all the OFT parameters NLC (F_3,12_ = 73.17, *p* < 0.001), CSE (F_3,12_ = 21.65, *p* < 0.001), DCS (F_3,12_ = 36.22, *p* < 0.001), and RR (F_3,12_ = 120.80, *p* < 0.001) significantly increased in all the dLAN treated groups in comparison to dLAN control groups in dose-dependent manner (Table 2). These findings substantiate curcumin associated improvement establishing that Cur150 is sufficient to positively modulate the locomotor activity and anxiety-like behaviour of mice.

### 3.2. Curcumin Improved the dLAN Induced Deterioration of Spatial and Retention Memory

Table 1 and Table 2 represent averages of Morris Water Maze (MWM) test parameters: namely, time spent to find platform (TSFP) and time spent in platform quadrant (TSPQ) in LD control, dLAN exposed, and dLAN treated (dLAN + Cur50, dLAN + Cur100, and dLAN + Cur150) mice. Results showed that the average value of TSFP (t = 22.47, *p* < 0.001) was significantly higher and that of TSPQ (t = 24.52, *p* < 0.001) was lower in mice exposed to dLAN in comparison to LD control group (Table 1). Thus, continuous exposure of mice to dLAN, impaired their spatial learning and retention memory. To modulate positively this impaired spatial and retention memory, mice of dLAN group were treated with increased concentrations (50, 100, and 150 mg/kg body weight) of curcumin. Results showed that both spatial and retention memory characterized by TSFP (F_3,12_ = 232.80, *p* < 0.001) and TSPQ (F_3,12_ = 194.70, *p* < 0.001) were positively modulated by curcumin in a dose-dependent manner (Table 2), establishing that curcumin is a significant modulator of hippocampal dependent spatial learning and memory functions.

### 3.3. Curcumin Reduced dLAN Induced Abrasion of Recognition Memory

Table 1 and Table 2 represent averages of novel object recognition (NOR) test parameter—namely, time spent with novel object/time spent with familiar object (T2/T1) in LD control, dLAN exposed, and dLAN treated (dLAN + Cur50, dLAN + Cur100, and dLAN + Cur150) mice. Results showed that the average value of T2/T1 ratio was significantly lower in mice exposed to dLAN (t = 29.04, *p* < 0.001) in comparison to LD control groups (Table 1). This suggests that dLAN exposure induces alteration of recognition memory in mice. To explore the positive modulatory effect of curcumin on recognition memory, mice of dLAN groups were treated with increasing concentrations (50, 100 and 150 mg/kg body weight) of curcumin. Results revealed that treatment of curcumin had significantly increased the recognition memory of dLAN (F_3,12_ = 227.30, *p* < 0.001) exposed mice in a dose-dependent manner (Table 2). This indicates that curcumin is an effective modulator of recognition memory of mice.

### 3.4. Curcumin Reduced dLAN Induced Lipid Peroxidation and Enhanced Superoxide Dismutase and Catalase Activity

Table 1 and Table 2 represent average values of malondialdehyde (MDA), superoxide dismutase (SOD), and catalase (CAT) activity in LD control, dLAN exposed, and dLAN treated (dLAN + Cur50, dLAN + Cur100, and dLAN + Cur150) mice. Results showed a significant difference in average levels of MDA (t = 17.45, *p* < 0.001), SOD (t = 24.32, *p* < 0.001), and CAT (t = 10.55, *p* < 0.001) activity between mice exposed to dLAN and LD control (Table 1). The level of MDA was higher, and SOD and CAT activity was lower, in mice exposed to dLAN as compared to LD control groups. This signifies that dLAN induces the lipid peroxidation through oxidative stress in mice. When the mice exposed to dLAN groups were treated with increasing concentrations of curcumin (50, 100, and150 mg/kg body weight), the average MDA (F_3,12_ = 167.40, *p* < 0.001) level was significantly decreased and SOD (F_3,12_ = 302.70, *p* < 0.001) and CAT (F_3,12_ = 101.00, *p* < 0.001) activity was increased in a dose-dependent manner as compared to dLAN exposed group (Table 2). The reduction in MDA level and the increase in SOD and CAT activity were more pronounced in mice treated with curcumin 150 mg/kg body weight. These findings indicate that curcumin confers protection against deleterious effects of dLAN exposure such as lipid peroxidation mediated oxidative stress.

### 3.5. Curcumin Reduced dLAN Induced Neuronal Abnormality

**Figure 3:**
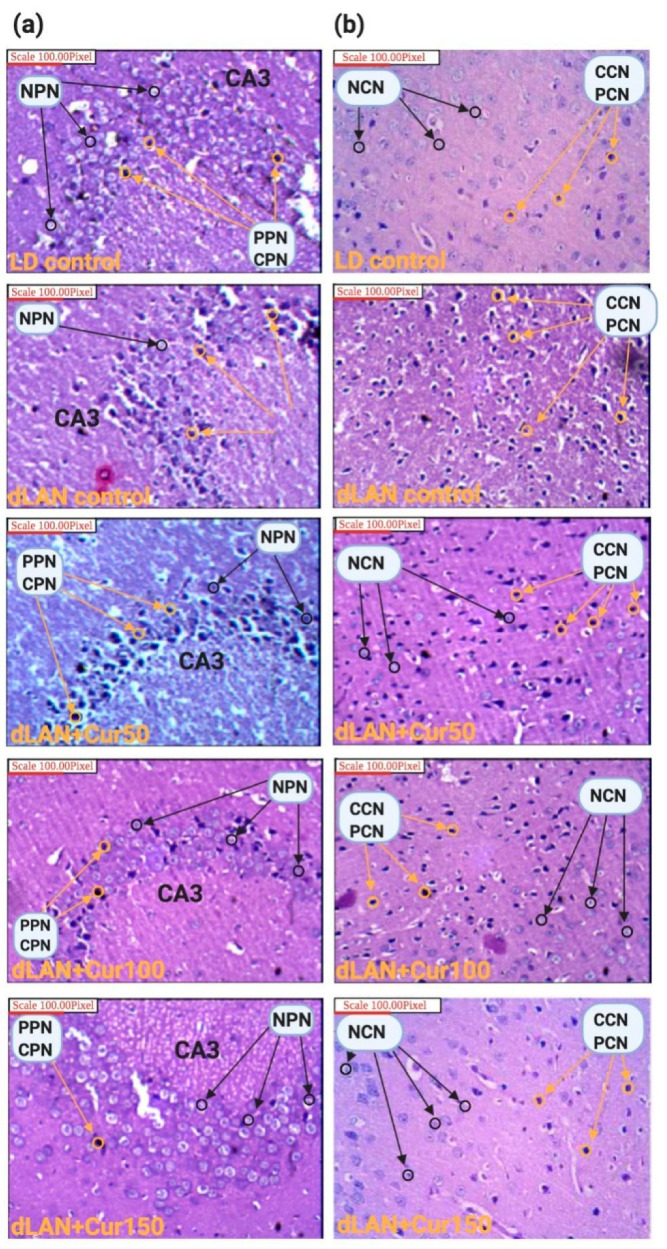
Effect of curcumin on LD and dLAN-exposed animals (**a**) hippocampal region showing CA3 pyramidal neurons (**b**) cerebral region showing cortex neurons. In dLAN exposed groups, higher numbers of pyknotic and chromatolysis neurons were seen in the hippocampal CA3 region as well as cortex region (shown by red arrows). Curcumin treatment had significantly reduced the pyknotic and chromatolysis neurons in a dose-dependent manner. More number of normal pyramidal and cortex neurons can be seen in the curcumin treated and LD groups (shown by black arrows). PPN: pyknotic pyramidal neurons; CPN: chromatolysis pyramidal neurons; PCN: pyknotic cortex neuron; CCN: chromatolysis cortex neuron; NPN: normal pyramidal neurons; NCN: normal cortex neurons; dLAN: dim light at night; LD: light (12 h)/dark (12 h).

Hematoxylin and eosin staining was used to study histopathological characteristics of hippocampal and cerebral regions of the mice brain. Results revealed severe degeneration of hippocampal CA3 pyramidal neurons and neurons of the cerebral region in mice exposed to dLAN. The number of pyknotic (irreversible condensation of chromatin in the cell nucleus leading to necrosis or apoptosis) and chromatolysis (dissolution of Nissl bodies) was more prominent in these regions of mice exposed to dLAN. Morphological disruption of hippocampal CA3 (Figure 3a) and cerebral cortex (Figure 3b) neurons were found to be effectively restored when the mice exposed to dLAN were treated with increased concentrations (50, 100, and 150 mg/kg body weight) of curcumin, indicating once again that a curcumin dose 150 mg/kg is a prominent one. These findings substantiate the neuroprotective effects of curcumin on the central nervous system.

### 3.6. Curcumin Up-Regulates the dLAN Induced Down-Regulation of Hippocampal BDNF, Synapsin II, and DCX Proteins in Dose Dependent Manner

**Figure 4:**
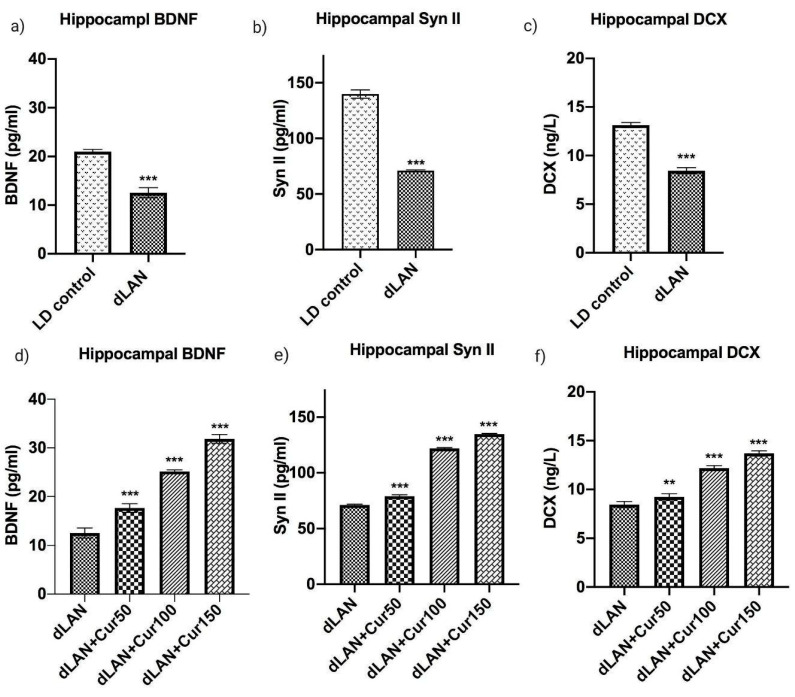
Effect of curcumin on dLAN altered hippocampal proteins (**a**,**d**) BDNF, (**b**,**e**) Synapsin II, and (**c**,**f**) DCX. Values are represented as mean ± standard deviation. The *t-*test was employed to compare the results between LD and dLAN exposed groups (**a**–**c**). Two-way ANOVA test was employed to compare the results between dLAN exposed vs. dLAN treated groups (**d**–**f**). All the data collected was analysed in GraphPad Prism-8 software. The values of ** *p* < 0.01, and *** *p* < 0.001 represents a statistically significant difference between the groups.

Figure 4 represents the mean levels of BDNF, Synapsin II and DCX proteins in the hippocampus homogenates in LD control, dLAN exposed and dLAN treated (dLAN + Cur50, dLAN + Cur100 and dLAN + Cur150) mice. When we compared the BDNF (t = 14.97, *p* < 0.001), Synapsin II (t = 36.33, *p* < 0.001) and DCX (t = 23.14, *p* < 0.001) protein levels between LD and dLAN mice, results showed significantly higher levels of these proteins in the LD control group (Figure 4a,c,e). This suggests that dLAN exposure had caused deleterious effects on neurogenesis through reduction of hippocampal neurogenesis associated proteins (as hypothesized and shown in Figure 1, right panel). In order to assess the effects of curcumin on the levels of these proteins, dLAN exposed mice were treated with increased concentrations of curcumin (50, 100 and 150 mg/kg body weight) and it was found that curcumin had increased the level of these proteins [BDNF (F_3,12_ = 416.30, *p* < 0.001), Synapsin II (F_3,12_ = 4831.00, *p* < 0.001), DCX (F_3,12_ = 286.60, *p* < 0.001)] in a dose-dependent manner (Figure 4b,d,f). Therefore, these findings suggest that curcumin is an effective modulator of hippocampal neurogenesis through regulation of hippocampal proteins (as hypothesized and shown in Figure 1, left panel).

### 3.7. Curcumin Enhanced the mRNA Levels of Hippocampal BDNF, Synapsin II, DCX, CREB and SIRT1 in Mice Exposed to dLAN

**Figure 5:**
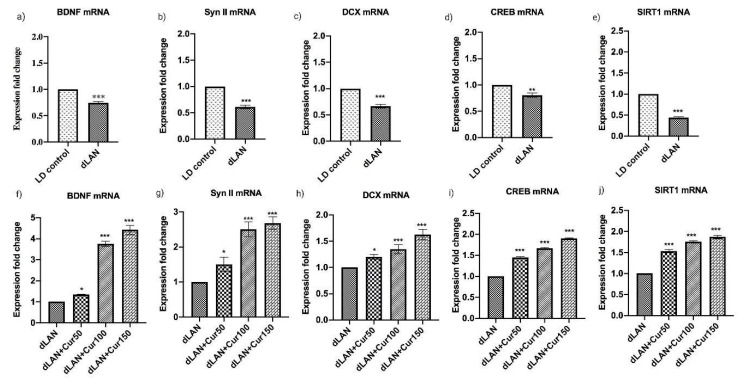
Effect of curcumin on dLAN altered hippocampal mRNA (**a**,**f**) BDNF, (**b**,**g**) Synapsin II, (**c**,**h**) DCX, (**d**,**i**) CREB, and (**e**,**j**) SIRT1. Values are represented as mean ± standard deviation. The *t-*test was employed to compare the results between LD and dLAN groups (**a**–**e**). Two-way ANOVA test was employed to compare the results between dLAN exposed vs. dLAN treated groups (**f**–**j**). All the data collected was analysed using GraphPad Prism-8 software. The values of * *p* < 0.05, ** *p* < 0.01, and *** *p* < 0.001 represents a statistically significant difference between the groups.

Figure 5a–j represents the mean mRNA expression levels of hippocampal BDNF, Syn II, DCX, CREB and SIRT1 (t = 46.02, *p* < 0.001) in LD control, dLAN exposed and, dLAN treated (dLAN + Cur50, dLAN + Cur100 and dLAN + Cur150) mice. Quantitative analysis revealed significantly higher mRNA levels of BDNF (t = 20.71, *p* < 0.001), Synapsin II (t = 19.29, *p* < 0.001), DCX (t = 16.51, *p* < 0.001), CREB (t = 8.27, *p* < 0.01) and SIRT1 (t = 46.02, *p* < 0.001) in LD control as compared to dLAN exposed groups (Figure 5a,c,e,g,i). This indicated that dLAN exposure effectively down-regulated mRNA expression of these proteins in mice (as hypothesized and shown in Figure 1, right panel). Further, when the mice in dLAN exposed group were treated with increasing concentrations (50, 100 and 150 mg/kg body weight) of curcumin, results showed a significant increase in BDNF (F_3,8_ = 588.40, *p* < 0.001), Synapsin II (F_3,8_ = 65.52, *p* < 0.001), DCX (F_3,8_ = 43.84, *p* < 0.001), CREB (F_3,8_ = 1672.00, *p* < 0.001) and SIRT1 (F_3,8_ = 609.10, *p* < 0.001) mRNA expression in dose-dependent manner and curcumin dose 150 mg/kg was found to be a prominent one (Figure 5b,d,f,h,j). These findings suggest that SIRT1 mediated CREB signalling pathway paves for the upregulation of BDNF, Synapsin II and DCX hippocampal proteins in the dLAN treated groups (as hypothesized and shown in Figure 1, left panel). This in turn improved the impaired and desynchronized behaviour in mice through regulation of hippocampal neurogenesis.

### 3.8. Influence of Curcumin on Altered Expression of Hippocampal miRNAs

**Figure 6:**
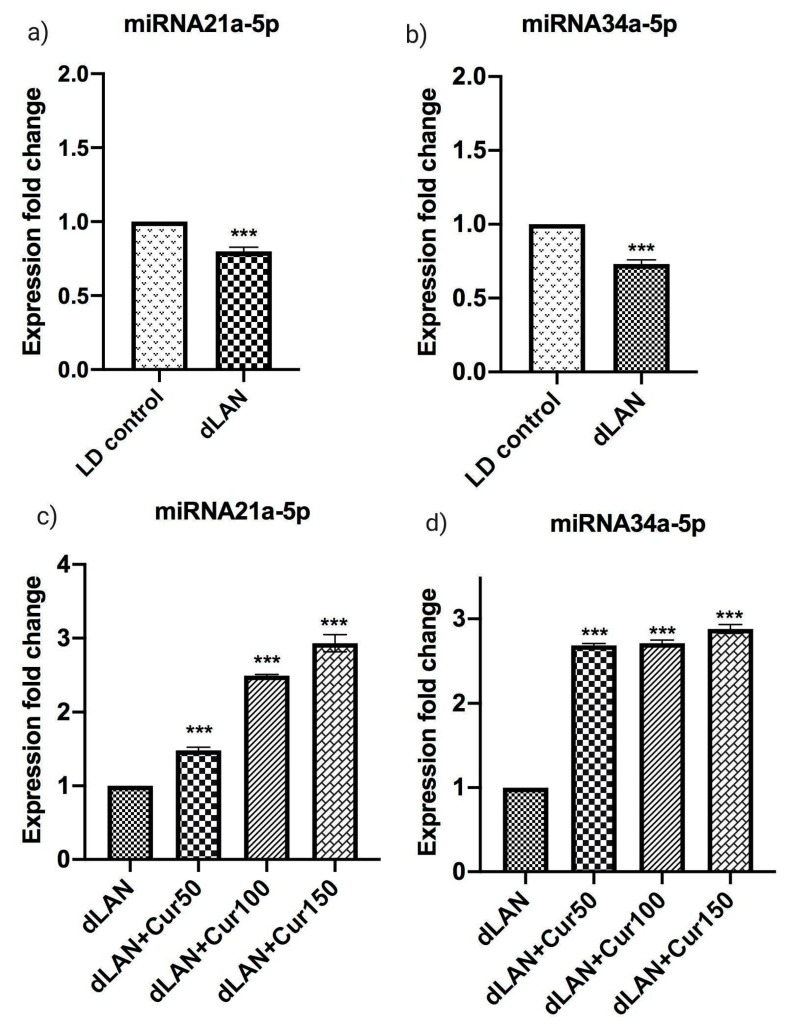
Effect of Curcumin on dLAN altered hippocampal miRNA (**a**,**c**) miRNA21a-5p, and (**b**,**d**) miRNA34a-5p. Values are represented as mean ± standard deviation. The *t-*test was employed to compare the results between LD and dLAN groups (**a**,**b**). Two-way ANOVA test was employed to compare the results between dLAN exposed vs. dLAN treated groups (**c**,**d**). All the data collected was analysed using GraphPad Prism-8 software. The values of *** *p* < 0.001 represents a statistically significant difference between the groups.

Figure 6a–d represents mean expression levels of miRNA21a-5p and miRNA34a-5p in LD control, dLAN exposed and dLAN treated (dLAN + Cur50, dLAN + Cur100 and dLAN + Cur150) mice. These two miRNAs are highly implicated in brain development and specially neurogenesis. When we compared the expression levels of miRNA21a-5p (t = 12.36, *p* < 0.001) and miRNA34a-5p (t = 16.91, *p* < 0.001) between LD control and dLAN groups, the expression of both miRNAs was found to be down-regulated in the dLAN exposed animals (Figure 6a,b, as hypothesized and shown in Figure 1, right panel). Further, when we compared the expression fold change of miRNA21a-5p (F_3,8_ = 596.10, *p* < 0.001) and miRNA34a-5p (F_3,8_ = 2023.00, *p* < 0.001) in the dLAN exposed mice with administration of different concentrations of curcumin, the results revealed that the expression of both miRNAs were significantly increased in curcumin treated mice in a dose-dependent manner (Figure 6c,d, (as hypothesized and shown in Figure 1, left panel). This implies that curcumin is an effective modulator of hippocampal miRNAs.

## 4. Discussion

Daily light/dark cycle controls the diurnal rhythm of the living organisms, which in turn regulates learning, memory and mood swings [2,4]. Disruption of diurnal rhythm has been attributed to certain environmental factors (for example shift work, air travel jet lag, and irregular food intake) and lifestyle of an individual. This has been found to be a major root cause of various pathological conditions including neurodegenerative disorders, depression, mood disorders, and cognitive impairment [13,14].

The data collected and analysed during our study highlights that dLAN exposure for three consecutive weeks has a detrimental effect on cognitive and non-cognitive behaviour, regulation of hippocampal genes (*BDNF, Synapsin II, DCX, CREB*, and *SIRT1*) and proteins (BDNF, Synapsin II, and DCX) and regulation of hippocampal miRNAs (miRNA21a-5p and miRNA34a-5p). In the present study, we hypothesized that dLAN induced detrimental effects in mice can be reversed by curcumin, which is an active polyphenol and has been known for its medicinal properties (Figure 1, left panel). Curcumin has been used in the treatment of oxidative and inflammatory conditions, exercise-induced inflammation and muscle soreness, arthritis, anxiety, metabolic syndrome and hyperlipidemia [51]. In addition, curcumin is known to be a target for multiple signalling pathways exemplifying its thrust as a potential therapeutic agent [28,29,30]. Moreover, administration of curcumin (50, 100, and 150 mg/kg) showed improvement in cognitive and non-cognitive behavioural of mice kept under normal light and dark cycle, possibly by promoting hippocampal neurogenesis (our published research [52], presented here as supplementary Figure S1). Therefore, in the present study, we have assessed the modulatory effects of curcumin on aforementioned parameters in dLAN exposed mice and found that treatment with curcumin effectively modulated all these parameters in a dose-dependent manner.

We observed a significant reduction in locomotor activity, spatial, retention and recognition memory and a significant increase in depressive-like behaviour in the dLAN exposed mice. The dLAN exposed mice were characterized by the lower number of lines crossed, centre square entries, duration in the centre square, and increased average number of rearings. Our findings are in accordance with the earlier studies where mice exposed to artificial dLAN condition were found to be highly susceptible to behavioural impairments and showed anxiety-like behaviour [53,54]. After administering curcumin in a dose dependent manner, the locomotor activity and depression-like behaviour improved in the dLAN exposed mice. Even though the curative effect of curcumin on dLAN induced behavioural impairment has not been studied thus far, several studies have highlighted that treatment with curcumin improved the cognitive and non-cognitive behaviours in rodents [36,39,40].

We found that dLAN exposed mice were associated with higher mean time spent to find the hidden platform (TSFP) and lower mean time spent in the platform quadrant (TSPQ). We inferred that dLAN exposure causes deterioration of spatial learning and retention memory function. Our observation is in accordance with other researchers who have reported dLAN induced impairment of spatial learning and retention memory function [20,54,55]. We observed that curcumin treatment caused improvement in spatial learning and retention memory function in the dLAN exposed mice when administered in a dose-dependent manner, where higher dose (150 mg/kg body weight) showed a higher degree of positive response.

The molecular and neuroanatomical mechanisms of cognitive impairment associated with dLAN exposure remain mostly unsolved. However, Taufique et al. [56] reported significant decrease in the neuroplasticity through the reduction of soma size and decreased number of glial cells in the hippocampus of Indian house crows exposed to dLAN. In the current study, we have also observed disruption and increased pyknosis and chromatolysis (characteristics of damaged neurons) in the hippocampal and cerebral cortex neurons of the dLAN exposed mice.

As we know, the growth and survival of newly produced neuronal cells in the hippocampal is dependent on the level of endogenous antioxidant (SOD, catalase) and pro-oxidant (MDA) level. We analysed the antioxidant levels in the hippocampus of the dLAN exposed mice and found a significant reduction in the enzymatic activity of SOD and catalase and elevation in the MDA levels. This suggests that the deterioration of hippocampal neuronal cells and the behaviour impairment of dLAN exposed mice could have been mediated by oxidative stress induced neuronal damage.

The extent of hippocampal neurogenesis depends on the micro-environment and there are many endogenously produced proteins which have essential roles in neuronal cell growth, differentiation, migration, maturation and integration in the existing pool of brain circuits [57]. Therefore, we investigated the expression levels of hippocampal proteins namely BDNF, Synapsin II and DCX in the dLAN exposed mice. The results revealed that the expression level of these proteins were significantly reduced in the dLAN exposed mice. Similar reports are there from other studies who mentioned the suppression of hippocampal neurogenesis through reduction of *DCX* and *BDNF* genes in the dLAN exposed animals [58].

Subsequently, we have analysed the expression pattern of the mRNAs of *BDNF, Synapsin II*, *DCX*, *CREB* and *SIRT1*. We have included *SIRT1* in our study as mammalian *SIRT1* has reported to possess multiple roles in brain development and cognitive performance. *SIRT1* deletion in mice brain leads to the reduction of cognitive task performance (spatial learning and memory) and neuroplasticity [59]. In our study, we observed significant reduction in the hippocampal *SIRT1* in the dLAN exposed mice. Therefore, the poor cognitive performance of mice exposed to dLAN might be due to the reduction of hippocampal neuroplasticity mediated through down-regulation of *SIRT1* mRNA. SIRT1 also upregulates brain BDNF expression through the activation of the CREB transcription pathway [60]. This suggested that the decreased BDNF and CREB proteins and mRNAs in the dLAN exposed mice could be mediated through reduction of hippocampal *SIRT1* expression.

These studies suggest that exposure to dLAN, which is quite common in the modern society has a significant effect on both cognitive and non-cognitive behaviour of animals through reduction of hippocampal neurogenesis. However, treatment with different curcumin concentrations had effectively restored the level of hippocampal proteins (BDNF, Synapsin II and DCX) and genes (*BDNF, Synapsin II*, *DCX*, *CREB*, and *SIRT1*) in a dose-dependent manner. This theory has been further supported by many researchers who have reported the upregulation of the hippocampal proteins in curcumin treated mice [61,62].

Further, we investigated the effects of dLAN exposure on the expression pattern of hippocampal miRNA21a-5p and miRNA34a-5p in mice. Many aspects of transcriptional/translational regulation of miRNAs contribute to the proper functioning of the centre and peripheral nervous system. MiRNAs regulate the expression of RNA molecules by binding to their 3′-untranslated region (UTR) [63,64]. Several miRNAs are reported to affect neurogenesis and neuroplasticity; disruption of their expression has led to several neurodegenerative diseases [65,66]. MiRNA21 is involved in the pathophysiology of neurodegenerative diseases [67,68]. MiRNA34a regulates the hippocampal neural stem cell (NSC) proliferation and further activation of the CREB pathway [69,70]. MiRNA34a activation is mediated by SIRT1 [71]. MiRNA34 depletion is implicated in the process of neurodegeneration and mitochondrial dysfunction [72]. Since the mitochondrial dysfunction and clearance of cellular waste are under the control of diurnal rhythm, the expression of miRNA21 and miRNA34 might be involved in the process of dLAN induced neurodegeneration. Our results revealed that the expression of both miRNAs is significantly reduced in the dLAN exposed mice. A feedback loop mechanism has been reported between SIRT1 and other miRNAs (including miRNA21) [71,73], downregulation of former leads to downregulation of latter and vice versa. The reduction of miRNAs in our study might be the indicator of reduced neurogenesis as miR34 helps in the proliferation of NSC and activation of CREB. Most of the genes and both the miRNAs studied by us are converging on the CREB pathway.

The downregulation of miRNA21 and miRNA34 in dLAN exposed mice suggests that the dLAN exposure caused the hippocampal neurodegeneration and reduction in neurogenesis possibly through the cross-talk among the SIRT1 and the miRNAs. However, treatment with increasing amounts of curcumin increased the expression of both hippocampal miRNAs in the dLAN exposed group and possibly increased neurogenesis. In the absence of such studies, we are unable to compare or corroborate our results.

Nevertheless, curcumin seems to be an effective therapeutic agent in the neurological disorders through regulation of hippocampal neurogenesis and improvement of behaviour of rodents.

Overall, the current study revealed that chronic exposure to dLAN has led to the degeneration of hippocampal neuronal cells through oxidative stress mediated by reduction of hippocampal neurogenesis associated genes (BDNF, Synapsin II, DCX, CREB, and SIRT1). The expression of BDNF and CREB seems to be interrelated, as the binding of BDNF to TrK-B receptor (present in the cell membrane) leads to auto-phosphorylation of the intracellular domain of the TrK-B receptor and activation of the downstream MAPK pathway. TrkB signalling activates the phosphatidylinositol-3 (PI3) kinase which stimulates cAMP response-element binding protein (CREB) which directs the biosynthesis of BDNF [34,74].

The reduction of BDNF protein and SIRT1 and its associated miRNAs expression led to down-regulation of the PI3K pathway and deactivation of the transcription factor CREB. The inactivation of PI3K and CREB pathways induced neurodegeneration and the consequences of behavioural impairment including a negative effect on learning and memory functions. However, treatment with curcumin increases the hippocampal neurogenesis through PI3K and CREB pathways, reduces the oxidative stress, leading to a behavioural improvement in mice, thus proving our hypothesis (Figure 1).

## 5. Conclusions

Several studies have discovered that dLAN exposure of animals exhibits a decline in spatial learning and memory performance, but the underlying mechanisms remain unknown. We have investigated the effect of dLAN exposure on the behaviour of mice based on cognitive and non-cognitive, histopathology, biochemical and molecular levels. The data assimilated by us and its subsequent analysis provides ample evidence that dLAN exposure leads to impairment in both cognitive and non-cognitive behaviour in mice through elevation of hippocampal oxidative stress and reduction of hippocampal neurogenesis. However, treatment with curcumin had effectively restored these parameters, when administered in a dose-dependent manner. The most effective dose was found to be 150 mg/Kg body weight. This work may be useful to cure jet lag of the frequent international flyers by modulating the light influx along with a prescribed dose of curcumin.

## Figures and Tables

**Table 1:** Alterations in the behavioural and biochemical parameters in control light–dark cycle (LD) and light/dim light (dLAN) cycle exposed groups.

Behavioural Parameters							
S.No.	Test	Parameters	Group		*t*-Test		
1			**LD control**	**dLAN exposed**	**t**	**df**	***p***
	OFT	NLC	152.75 ± 5.50 ^a^	97.25 ± 1.47 ^b^	19.27	6	*p* < 0.001
		CSE (n)	06.00 ± 0.81 ^a^	3.45 ± 0.42 ^b^	5.549	6	*p* < 0.01
		CSD (s)	12.48 ± 0.55 ^a^	7.91 ± 0.58 ^b^	11.45	6	*p* < 0.001
		RR (n)	45.25 ± 1.70 ^a^	23.25 ± 1.50 ^b^	19.36	6	*p* < 0.001
2	MWM	TSFP (s)	42.25 ± 0.95 ^a^	64.25 ± 1.70 ^b^	22.47	6	*p* < 0.001
		TSPQ (s)	49.50 ± 1.29 ^a^	23.25 ± 1.70 ^b^	24.52	6	*p* < 0.001
3	NOR	T2/T1	1.24 ± 0.01 ^a^	0.48 ± 0.05 ^b^	29.04	6	*p* < 0.001
**Biochemical Parameters**							
4	MDA (nM/mg)		2.10 ± 0.01 ^a^	3.17 ± 0.05 ^b^	17.45	6	*p* < 0.001
5	SOD (unit/mg)		6.46 ± 0.31 ^a^	2.44 ± 0.22 ^b^	24.32	6	*p* < 0.001
6	CAT (unit/mg)		3.37 ± 0.08 ^a^	2.33 ± 0.08 ^b^	10.55	6	*p* < 0.001

The non-parametric *t-*test was used to compare the results between LD and dLAN groups. Mean values bearing the dissimilar alphabets are statistically different at group level (*p* < 0.001).OFT = open field test; MWM = Morris Water Maze; NOR = novel object recognition; NLC = Number of line cross; CSE = centre square entry; CSD = centre square duration; RR = rearing; TSFP = time spent to find platform; TSPQ = time spent in platform quadrant; T2/T1 = time spent with novel object/time spent with familiar object; MDA = malondialdehyde; SOD = superoxide dismutase; CAT = catalase; n = number; s = second.

**Table 2:** Effect of curcumin on dLAN induced behavioural and biochemical alterations in mice.

Behavioural Parameters									
S.No.	Test	Parameters	Group				One-Way ANOVA		
1	OFT		**dLAN**	**dLAN + Cur50**	**dLAN + Cur100**	**dLAN + Cur150**	**F**	**df**	***p***
		NLC (n)	97.25 ± 1.47	107.75 ± 1.70 ^a^	120.00 ± 4.39 ^b^	124.25 ± 2.75 ^c^	73.17	12	*p* < 0.001
		CSE (n)	3.45 ± 0.42	4.50 ± 0.57 ^a^	5.50 ± 0.57 ^b^	6.25 ± 0.5 ^c^	21.65	12	*p* < 0.001
		CSD (s)	7.91 ± 0.58	8.11 ± 0.43	9.56 ± 0.45	14.37 ± 1.81 ^a^	36.22	12	*p* < 0.001
		RR (n)	23.25 ± 1.50	30.00 ± 1.82 ^a^	39.00 ± 1.41 ^b^	40.25 ± 0.95 ^c^	120.80	12	*p* < 0.001
2	MWM	TSFP (s)	64.25 ± 1.70	58.39 ± 0.74 ^a^	55.87 ± 1.39 ^b^	42.76 ± 0.50 ^c^	232.80	12	*p* < 0.001
		TSPQ (s)	23.25 ± 1.70	26.41 ± 0.72 ^a^	32.50 ± 1.53 ^b^	44.82 ± 1.27 ^c^	194.70	12	*p* < 0.001
3	NOR	T2/T1	0.48 ± 0.05	0.63 ± 0.018 ^a^	0.94 ± 0.04 ^b^	1.19 ± 0.04 ^c^	227.30	12	*p* < 0.001
**Biochemical Parameters**									
4	MDA (nM/mg)		3.17 ± 0.0476	2.66 ± 0.09 ^a^	1.89 ± 0.14 ^b^	1.52 ± 0.09 ^c^	167.40	12	*p < 0.001*
5	SOD (unit/mg)		2.44 ± 0.219	3.56 ± 0.10 ^a^	4.74 ± 0.18 ^b^	5.55 ± 0.21 ^c^	302.70	12	*p < 0.001*
6	CAT (unit/mg)		2.33 ± 0.08	2.64 ± 0.06	3.04 ± 0.11 ^a^	3.82 ± 0.13 ^b^	101.00	12	*p < 0.001*

Non-parametric one-way ANOVA with a posthoc Dunken’s test was employed to compare the behavioural results between dLAN exposed vs. dLAN treated groups. Two-way ANOVA test was employed to compare the biochemical result between dLAN exposed vs. dLAN treatment group. Mean values bearing the dissimilar alphabets are statistically different at group level (*p* < 0.001).OFT = open field test; MWM = Morris Water Maze; NOR = novel object recognition; NLC = number of line cross; CSE = centre square entry; CSD = centre square duration; RR = rearing; TSFP = time spent to find platform; TSPQ = time spent in platform quadrant; T2/T1 = time spent with novel object/time spent with familiar object; MDA = malondialdehyde; SOD = superoxide dismutase; CAT = catalase; n = number; s = second.

## Supplementary Materials

The following are available online at https://www.mdpi.com/2073-4409/9/9/2093/s1, **Figure S1:** Effect of curcumin on hippocampal protein level, **Table S1:** Details of experimental design, **Table S2:** Quantitative/real time PCR primer pairs, **Table S3:** Quantitative/real time PCR primer for microRNA.
