# Supplementary material for: Dim Light at Night Induced Neurodegeneration and Ameliorative Effect of Curcumin
Source: Cells. 2020 Sep 13;9(9):2093. doi: 10.3390/cells9092093 (PMC7565558; doi:10.3390/cells9092093)
Supplement: Supplementary file 1 [file cells-09-02093-s001.zip › cells-897995-SI/Supplementary Figure 1.pdf]

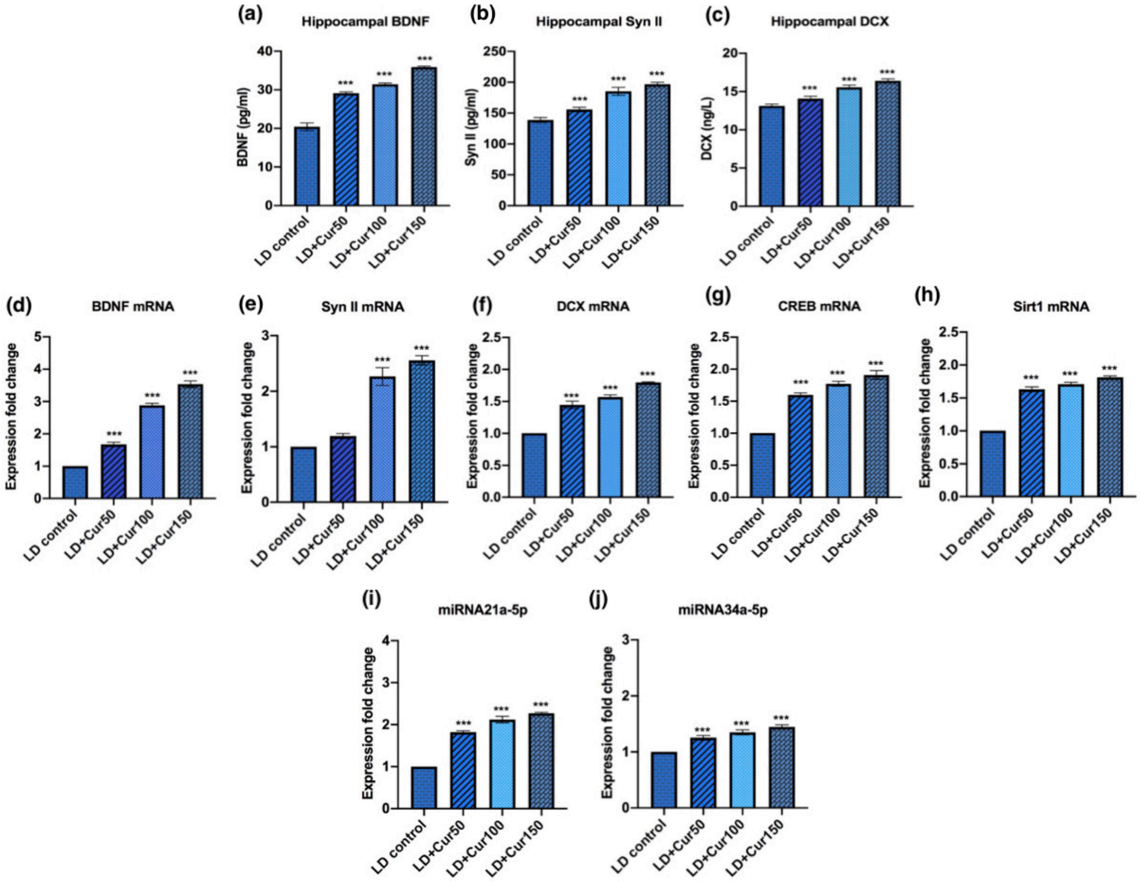

Figure S1: Effect of curcumin on hippocampal protein level (a) BDNF, (b) Synapsin II, (c) DCX; mRNA expression level (d) BDNF mRNA, (e) Synapsin II mRNA, (f) DCX mRNA, (g) CREB mRNA, (h) SIRT1 mRNA; and miRNA expression level (i) miRNA21a-5p and (j) miRNA34a-5p. Values are represented as mean ± standard deviation (n=7). One Way ANOVA with a posthoc-Dunken's test was employed to compare the results between control vs curcumin-treated groups. The data were analyzed using Graphpad Prism-8. The values *** $P<0.001$ represent a statistically significant difference between the groups.
