# Supplementary material for: Dim Light at Night Induced Neurodegeneration and Ameliorative Effect of Curcumin
Source: Cells. 2020 Sep 13;9(9):2093. doi: 10.3390/cells9092093 (PMC7565558; doi:10.3390/cells9092093)
Supplement: Supplementary file 1 [file cells-09-02093-s001.zip › cells-897995-SI/Supplementary table 1.pdf]

Table S1. Details of experimental design.

S. No.	Groups	Number of mice(n)	Experiment conditions	Treatment	Duration
1	**LD control**	6	12:12 light [~150 lux]/dark [~0 lux]	1%CMC	3 weeks
2	**dLAN control**	6	12:12 light [~150 lux]/dim light [~5 lux]	1%CMC	3 weeks
3	**dLAN+Cur50**	6	12:12 light [~150 lux]/dim light [~5 lux]	50mg/kg curcumin	3 weeks
4	**dLAN+Cur100**	6	12:12 light [~150 lux]/dim light [~5 lux]	100mg/kg curcumin	3 weeks
5	**dLAN+Cur150**	6	12:12 light [~150 lux]/dim light [~5 lux]	150mg/kg curcumin	3 weeks
