# Supplementary material for: Dim Light at Night Induced Neurodegeneration and Ameliorative Effect of Curcumin
Source: Cells. 2020 Sep 13;9(9):2093. doi: 10.3390/cells9092093 (PMC7565558; doi:10.3390/cells9092093)
Supplement: Supplementary file 1 [file cells-09-02093-s001.zip › cells-897995-SI/Supplementary table 2.pdf]

Table S2. Quantitative/real time PCR primer pairs

S.No.	Gene	Forward primer (5'-3')	Reverse primer (5'-3')
1	BDNF	GAAGAGCTGCTGGATGAGGAC	TTCAGTTGGCCTTTGGATACC
2	Synapsin II	TCAGCAAGATGAACCAGC	GACTTGTTGAGCTGTGGG
3	DCX	TGCTCAACCCAGAGAGAACA	CTGCTTTCCATCAAGGGTGT
4	CREB	CCAAACTAGCAGTGGGCAGT	GAATGGTAGTACCCGGCTGA
7	Sirt1	AGTTCCAGCCGTCTCTGTGT	CTCCACGAACAGCTTCACAA
8	β-actin	TGGTGGGTATGGGTCAGAAGGACTC	CATGGCTGGGGTGTTGAAGGTCTCA
