# Supplementary material for: Dim Light at Night Induced Neurodegeneration and Ameliorative Effect of Curcumin
Source: Cells. 2020 Sep 13;9(9):2093. doi: 10.3390/cells9092093 (PMC7565558; doi:10.3390/cells9092093)
Supplement: Supplementary file 1 [file cells-09-02093-s001.zip › cells-897995-SI/Supplementary table 3.pdf]

**Table S3.** Quantitative/real time PCR primer for microRNA

| **S.No.** | **microRNA Name** | **Forward primer (5'-3')** | **Reverse primer (5'-3')** |
|---|---|---|---|
| 1 | U6 | GCTTCGGCAGCACATATACTAAAAT | CGCTTCACGAATTTGCGTGTCAT |
| 2 | miR-34a-5p | GGTGGCAGTGTCTTAGCT | CAGTGCGTGTCGTGGAGT |
| 3 | miR-21a-5p | GGGGGGTAGCTTATCAGACTG | CAGTGCGTGTCGTGGAGT |
